# Phylogenetic Signal Dissection Identifies the Root of Starfishes

**DOI:** 10.1371/journal.pone.0123331

**Published:** 2015-05-08

**Authors:** Roberto Feuda, Andrew B. Smith

**Affiliations:** 1 Division of Biology and Biological Engineering, California Institute of Technology Pasadena, California, United States of America; 2 Department of Earth Sciences, The Natural History Museum, London, United Kingdom; BiK-F Biodiversity and Climate Research Center, GERMANY

## Abstract

Relationships within the class Asteroidea have remained controversial for almost 100 years and, despite many attempts to resolve this problem using molecular data, no consensus has yet emerged. Using two nuclear genes and a taxon sampling covering the major asteroid clades we show that non-phylogenetic signal created by three factors - Long Branch Attraction, compositional heterogeneity and the use of poorly fitting models of evolution – have confounded accurate estimation of phylogenetic relationships. To overcome the effect of this non-phylogenetic signal we analyse the data using non-homogeneous models, site stripping and the creation of subpartitions aimed to reduce or amplify the systematic error, and calculate Bayes Factor support for a selection of previously suggested topological arrangements of asteroid orders. We show that most of the previous alternative hypotheses are not supported in the most reliable data partitions, including the previously suggested placement of either Forcipulatida or Paxillosida as sister group to the other major branches. The best-supported solution places Velatida as the sister group to other asteroids, and the implications of this finding for the morphological evolution of asteroids are presented.

## Introduction

**Fig 1:**
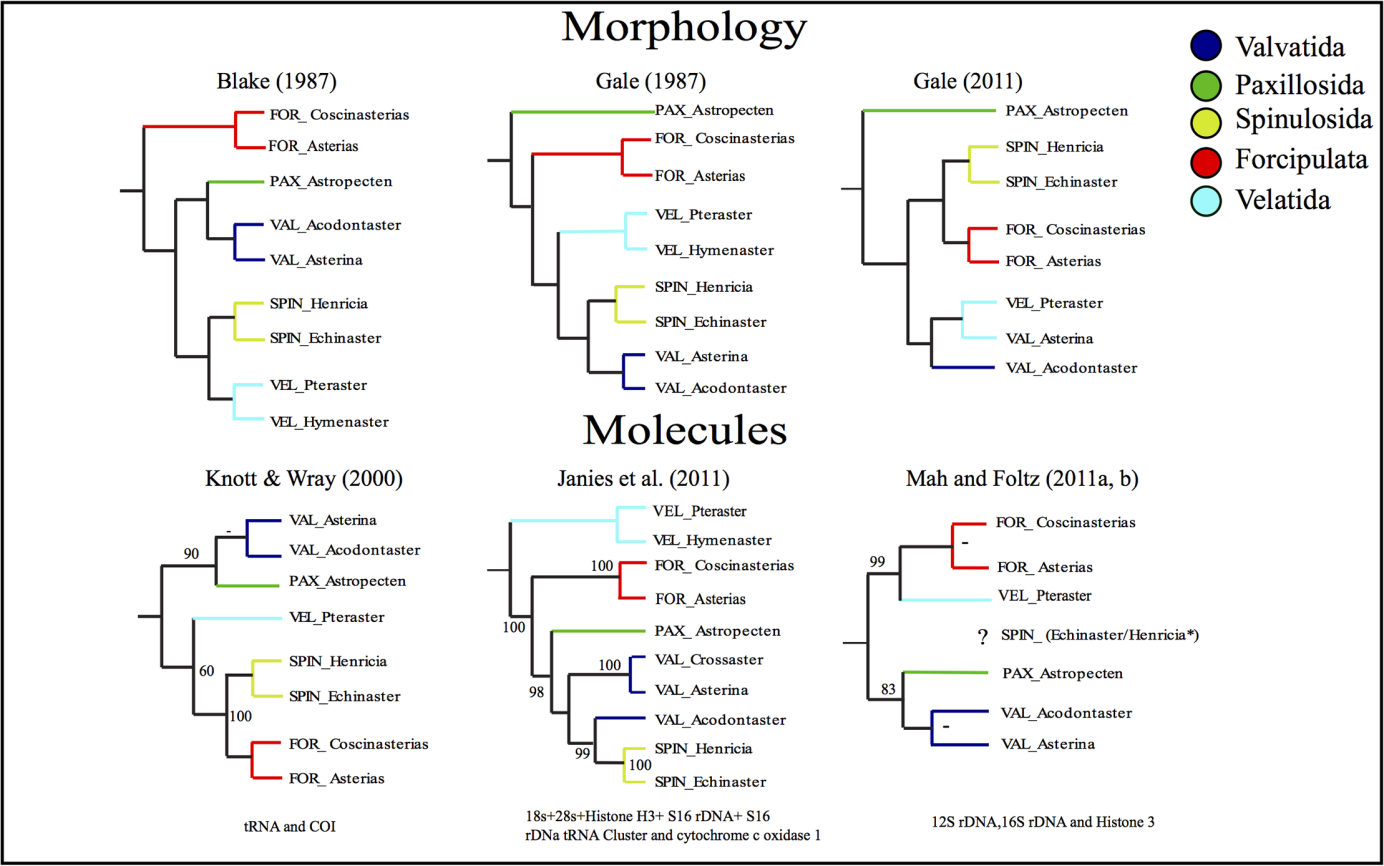
Current competing hypotheses of relationships derived from morphological or molecular data for the major starfish clades considered in this paper.

Starfishes (Asteroidea) are a morphologically well-defined clade, the most iconic of the five extant classes of echinoderm. This group includes around 1900 extant species classified into five major orders: Paxillosida, Spinulosida, Velatida, Valvatida and Forcipulata [1]. While there has never been any doubt about the monophyly of the crown group from a morphological [2–5] or molecular [6–9] perspective, relationships among the orders are far from settled. Disagreement continues in particular about how the crown group should be rooted and, consequently, the relationships of the various orders. This argument started in 1921 with the debate between Mortensen and MacBride [10–11], and ignited again in 1987 when two morphology-based phylogenies of the Asteroidea were published that came to very different conclusions (Fig 1). Blake [4] identified the order Forcipulatida as sister group to other asteroids whereas Gale [2] followed traditional interpretations placing Paxillosida in that position. Since then Blake [5, 12], Gale [3] and others [13] have continued to debate the relative merits of each interpretation from a morphological perspective.

Given this striking disagreement amongst morphologists, various attempts have been made to resolve asteroid relationships using molecular data [7–8, 14–19]. Unfortunately these too have failed to arrive at a consistent answer and, depending upon taxa included, genes analysed and method of analysis employed, have identified a variety of possible taxa at the base of crown group asteroids (Fig 1). Initial analyses based on small data sets [14–15] identified Paxillosida as sister group to other asteroids. However, Knott and Wray [17] using two mitochondrial genes found neither paxillosids nor forcipulatids as basal, instead placing a paraphyletic valvatids basal with velatids nested within this grade. Janies’ [7] combined morphological and molecular investigation of echinoderm class relationships included 16 asteroid genera. Although not specifically examining asteroid rooting this presented a fourth topology again with valvatids as paraphyletic but with velatids as basal. A later analysis [8], again focusing on class relationships but including 35 asteroids and partial sequences from 7 genes, identified a clade comprising two velatids (*Pteraster* and *Hymenaster*) and the highly divergent *Xyloplax* as sister group to other asteroids with Forcipulatida as the next major clade to branch after that. The recent extensive studies of asteroid relationships by Mah & Foltz [18–19] grouped Velatida with Forcipulatida and Paxillosida with Valvatida but left the Spinulosida unplaced. Thus both the branching pattern and the root position of the asteroid tree remains disputed and Fig 1 summarises the major competing topologies that have been proposed.

There are many potential reasons why molecular data have generated different results, as each analysis has used different approaches applied to different suites of taxa and gene sequences of varying completeness. Significantly, none have seriously attempted to correct for potential systematic bias in their data. It is now widely recognized that non-phylogenetic signal is a common problem that can distort molecular phylogenies [20– 21]. Non-phylogenetic signal has multiple and disparate sources. The use of suboptimal models of evolution, missing data [22], the presence of fast evolving taxa and compositional heterogeneity are all widely recognized as potential sources of false signal, with the latter two effects causing sequences to be erroneously grouped according to their relative branch lengths or analogous nucleotide or amino acid composition [23– 24]. Here we apply a suite of newly developed statistical techniques to a data set that includes representatives of the major starfish clades in order to study the effect of non-phylogenetic signal on our perception of starfish relationships.

## Methods and Materials

### Choice of taxa and genes

Representative species from each of the five major asteroid clades were selected: *Astropecten* (Paxillosida); *Asterias* and *Coscinasterias* (Forcipulatida); *Henricia* and *Echinaster* (Spinulosida); *Asterina* and *Acodontaster* (Valvatida) and *Pteraster* and *Hymenaster* (Velatida)—classification follows [18–19]. As the statistical power of our approach improves with larger data sets and missing data could generate artifacts [22], we selected the two representatives with the most complete gene sequences in each clade. Two orders, Brisingida and Cocentricycloidea, each encompassing relatively few species, could not be included in our analyses as gene sequence data for these deep-sea taxa was very incomplete. A selection of hemichordates and representatives of the four other echinoderm classes were included as outgroups (Table A in S1 File). Sequence data were assembled for two nuclear ribosomal genes (18S and 28S rRNA) and two mitochondrial ribosomal genes (12S and 16S rRNA). However, applying posterior probability analysis, as implemented in Phylobayes [25], we found that the amount of homoplasy was significantly higher in the combined dataset than in the dataset of nuclear genes (Table D in S1 File). Consequently, mitochondrial ribosomal genes were excluded from the analysis because of the high levels of saturation they displayed. S1 Dataset lists taxa and the sequences used. After alignment any regions that could not be unambiguously aligned across both ingroup and outgroup were deleted. The final data set comprises 31 species and 3017 positions and is available as Electronic Supplementary Data.

### Phylogenetic analysis

We first identified the best fitting model using a 12-fold Bayesian Cross-validation as implemented in Phylobayes 3.3e [25]. We compared the site-heterogeneous models CAT-GTR- Γ and CAT- Γ versus the site homogeneous model GTR-Γ. Results of the cross validation (Table B in S1 File) suggest that CAT-GTR-Γ generates the best fit. Accordingly our phylogenetic reconstruction for the full data set was performed using Bayesian Analysis under the optimal CAT-GTR- Γ model and the worst performing GTR-Γ model using Phylobayes3.3e [25]. Among site rate variation was modeled using a discrete Gamma distribution (4 rate categories). For all Phylobayes analyses two runs were performed and convergence was investigated using the bpcomp option (part of the Phylobayes package). Phylogenetic reconstruction was performed using maximum likelihood methods as implemented in PhyML under GTR-Γ. Node supports were evaluated using aBayes [26].

### Phylogenetic signal dissection and compositional heterogeneity

Phylogenetic signal dissection [27] was performed to assess the effect of fast evolving sites on tree topology. Site-specific rates of evolution were estimated using the program TIGER [28], which assigns evolutionary rates to characters and places them in bins of approximately equal rate. This is tree independent, eliminating the need for *a priori* tree specification [28]. Two partitions were created: the first containing the fastest evolving sites plus invariant sites (our ‘heterogeneous rates partition’- 2284 positions); the second contained all the remaining variant sites (our ‘homogeneous rates partition’- 733 positions). Because the first data partition combines sites with extreme rate variation this partition poses an extreme problem for tree reconstructing methods and is expected to be misled more readily by systematic bias [29].

The presence of compositional heterogeneity was evaluated using posterior predictive analysis (PPA) as implemented in Phylobayes [25]. As composition heterogeneity was found to be an important issue for the Asteroidea (see Table C in S1 File) we applied two approaches to minimize the problem. First we performed the phylogenetic reconstruction of the full data set using the non-stationary CAT-break point model (CATBP) [30], as implemented in Nh-phylobayes [30]. This model, by implementing a multiple break point along the lineages, is able to account directly for compositional heterogeneity [30]. Among site variation was modeled using four discrete gamma categories. Convergence among chains was evaluated using compchain software, which is part of the Nh-phylobayes package. Additionally we performed site stripping as implemented in the program BMGE [31]. BMGE used a Stuart’s test of marginal homogeneity to remove compositionally heterogeneous sites. This removed 278 heterogeneous sites from our original data and generated a partition contain only compositionally homogeneous sites.

As heterogeneity of both rates and composition has been identified as potential problems, we generated a final partition removing the heterogeneous compositional sites from the homogenous rates partition (our ‘composition+rate homogeneous partition’- 556 positions). This data set comprises the best set of sites with a *bona-fide* phylogenetic signal. Since that performance of complex model such as CAT-GTR seems to be less efficient on small alignment (less then 1000 position—[32]) we decided to analyse all subpartitions under GTR-Γ using Phylobayes3.3e. However, CAT-GTR-Γ was used to confirm the topology obtained using the most reliable composition+rate homogeneous partition.

The overall quality of the partition was evaluated using statistical criteria (see Table C in S1 File) and biological criteria (Table 1). While the phylogenetic relationships amongst asteroid taxa remain uncertain, some relationships in other echinoderm classes are securely founded, supported by both morphological and molecular data. The position of cidaroids (*Calocidaris*, *Stereocidaris*) as sister group to euechinoids (*Arbacia*, *Paracentrotus* and *Strongylocentrotus*) is unambiguously supported [33–34], as is the sister group relationship of Euryalida (*Asteronyx*, *Gorgonocephalus*) to Ophiurida (*Ophioderma*, *Ophiopsammus*, *Ophiocoma*, *Ophiothrix*, *Ophiopholis*) [35]. Similarly both morphological and molecular data place Elasipoda (*Psychropotes*) as sister group to the Aspidochirotida (*Cucumaria*, *Holothuria*) [36–37]. The relationships amongst some of the five classes of echinoderms are also largely uncontroversial, with crinoids as sister group to the other four classes and holothurians and echinoids as sister groups [29, 38]. We used these relationships as an additional criterion: data and methods that do not recover these well-founded relationships cannot be relied upon to have identified the correct root position for asteroids. Conversely, only when our data and methods identify these clades can we start to have faith in the root position identified for asteroids.

**Table 1:** This table summarizes the ability of the various partitions to identify well-established relationship amongst echinoderms other than the Asteroidea.

	Class relations	Echinoids	Ophiuroids	Holothurians	Crinoids
CAT-GTR-full	√	x	x	√	√
CAT-BP	√	√	√	√	√
GTR-full	√	√	√	√	√
GTR hetero rates	x	x	x	x	√
GTR homo comp	√	√	√	√	√
GTR homo rates	√	√	√	√	√
CAT-GTR, GTR rates+comp homo	√	√	√	√	√

X = the partition failed to recover the expected topology within the clade indicated; √ = the partition identified the expected topology within the clade indicated.

### Testing support for competing hypotheses

**Fig 2:**
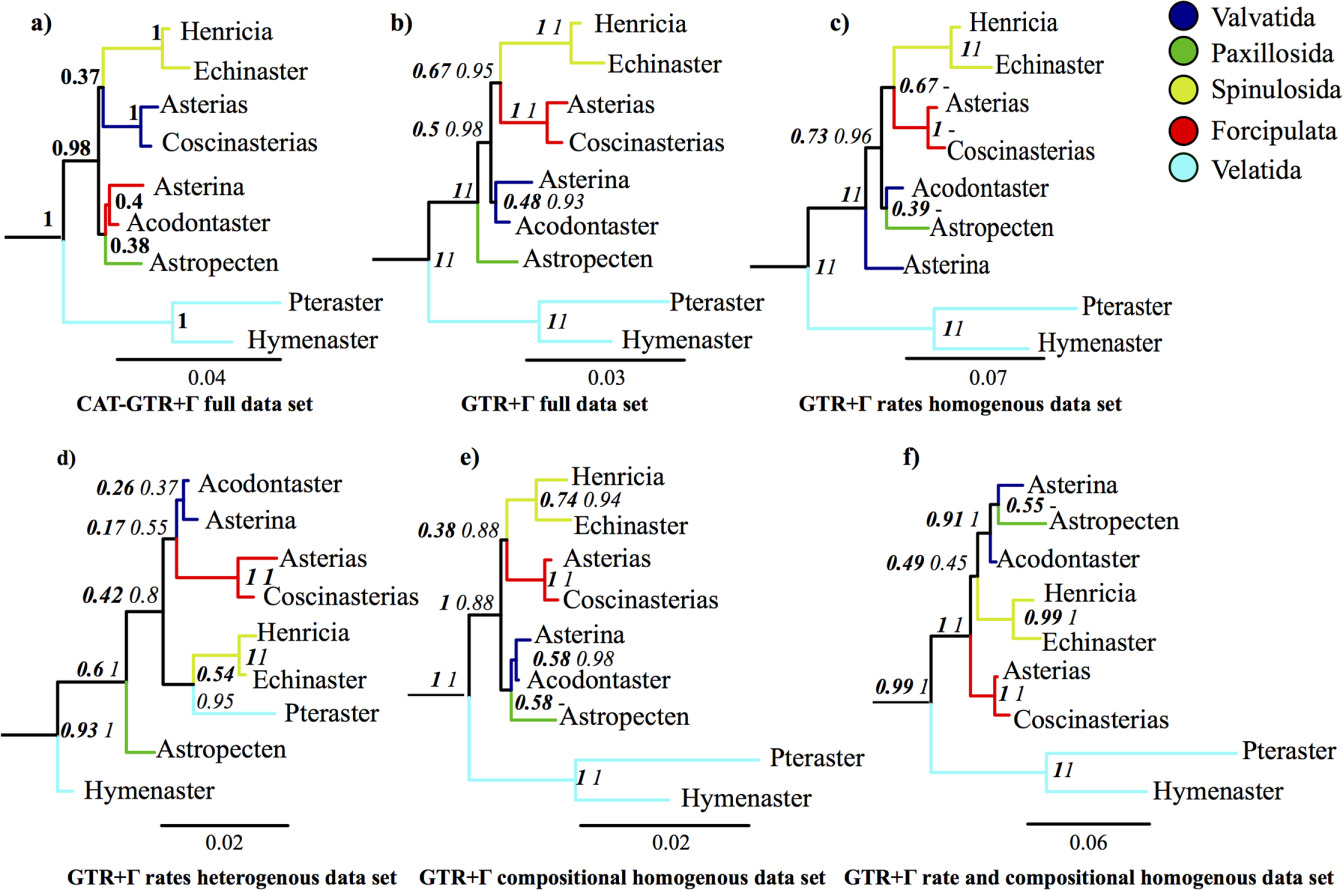
Cladograms summarizing inferred asteroid relationships obtained from the different partitions and under different substitution models. Numbers at nodes indicate Bayesian posterior probability support under CAT-GTR- Γ (bold), Bayesian posterior probability under GTR- Γ (italic bold) and maximum likelihood abayes bootstrap under GTR- Γ (italic). For all the trees except 2a, the branch lengths are estimated under Bayesian GTR- Γ.

Support amongst competing hypotheses was assessed using a Bayes Factor (BF) approach [39]. The marginal likelihood for each constraint tree was estimated under GTR-Γ using the stepping-stone procedure, as implemented in MrBayes3.2 (with 10 million generations and sampling every 2500 generations). The stepping-stone method has recently been developed and is known to outperform the traditional harmonic mean approach for estimating the marginal likelihood support [40]. We also compared the support for each of the topologies shown in Fig 1 (excluding [1], because of its incomplete coverage of asteroids orders) and Fig 2, in each data partition.

## Results

The data partitions we generated differ markedly in their composition homogeneity and saturation (Table C in S1 File) and in their ability to recover well-founded relationships within Echinoderms (Table 1). Furthermore, analysis of the same data partition under different evolutionary models (GTR vs. CAT-GTR) also generates different topologies (Fig 2). Two points emerge clearly from these analyses. First, there is strong support for Velatida at the root of Asteroidea that is rarely masked by non-phylogenetic signal. Second, the relationships among the remaining orders are partition dependent and any surviving phylogenetic signal is weak.

The support for Velatida as sister group to other Asteroidea has the highest posterior probability (PP = 1) in all the reliable partitions and under the best fitting model (Fig 2A, 2B, 2C, 2E and 2F and Figs S1–S4, S6 and S7). Only the least reliable rate heterogeneous partition does not support this topology, instead suggesting that Velatida are paraphyletic with one species monophyletic with Spinulosida (PP = 0.95) (Fig 2D and S5 Fig). The same pattern is observed in the maximum likelihood trees (Figs S8 – S12).

Regarding the relationships among starfishes other than Velatida, our results are partition and model dependent (Fig 2 and Figs S1–S12). Analysis of the full data set under the GTR-Γ model finds a poorly supported topology where Forcipulata and Spinulosida together form a monophyletic group (PP = 0.67), the Valvatida are monophyletic, but without support (PP = 0.48), and Paxillosida lie nested between the Velatida and all other starfishes (PP = 0.5). The same partition analysed under CATBP-Γ (which takes account of compositional heterogeneity—see Table C in S1 File) suggests a polytomy for Spinulosida and Forcipulata (PP<0.5) and the paraphyly of Valvatida, with Paxillosida nested within this group (PP = 0.7) (S3 Fig). Finally, when the best fitting CAT-GTR- Γ model is used all nodes, with the exception of the root position of Velatida, have insignificant support (Fig 2A).

Focusing on the more reliable data partitions only, the main differences we observe concern the level of support for the monophyly of Forcipulata and Spinulosida (ranging from PP = 0.67 in the rate-homogeneous partition (Fig 2C and S4 Fig) to PP = 0.38 in the composition-homogeneous partition (Fig 2E and S6 Fig), and the placement of the Paxillosida, which either nests within the Valvatida (Fig 2E and S6 Fig; PP = 0.58) or lies between Velatida and the other starfishes, as in the full data set (PP = 0.5, Fig 2B and S2 Fig). However, our composition+rates homogeneous data set, under Bayesian CAT-GTR- Γ, GTR- Γ and maximum likelihood GTR- Γ (Fig 4, S7 Fig and S12 Fig) suggests a different topology where Valvatida, Forcipulata and Spinulosida are monophyletic, albeit without support (PP = 0.49), Paxillosida and Valvatida form a monophyletic group (PP = 0.91) and Forcipulata now nests between Velatida and all the other Asteroidea.

**Fig 3:**
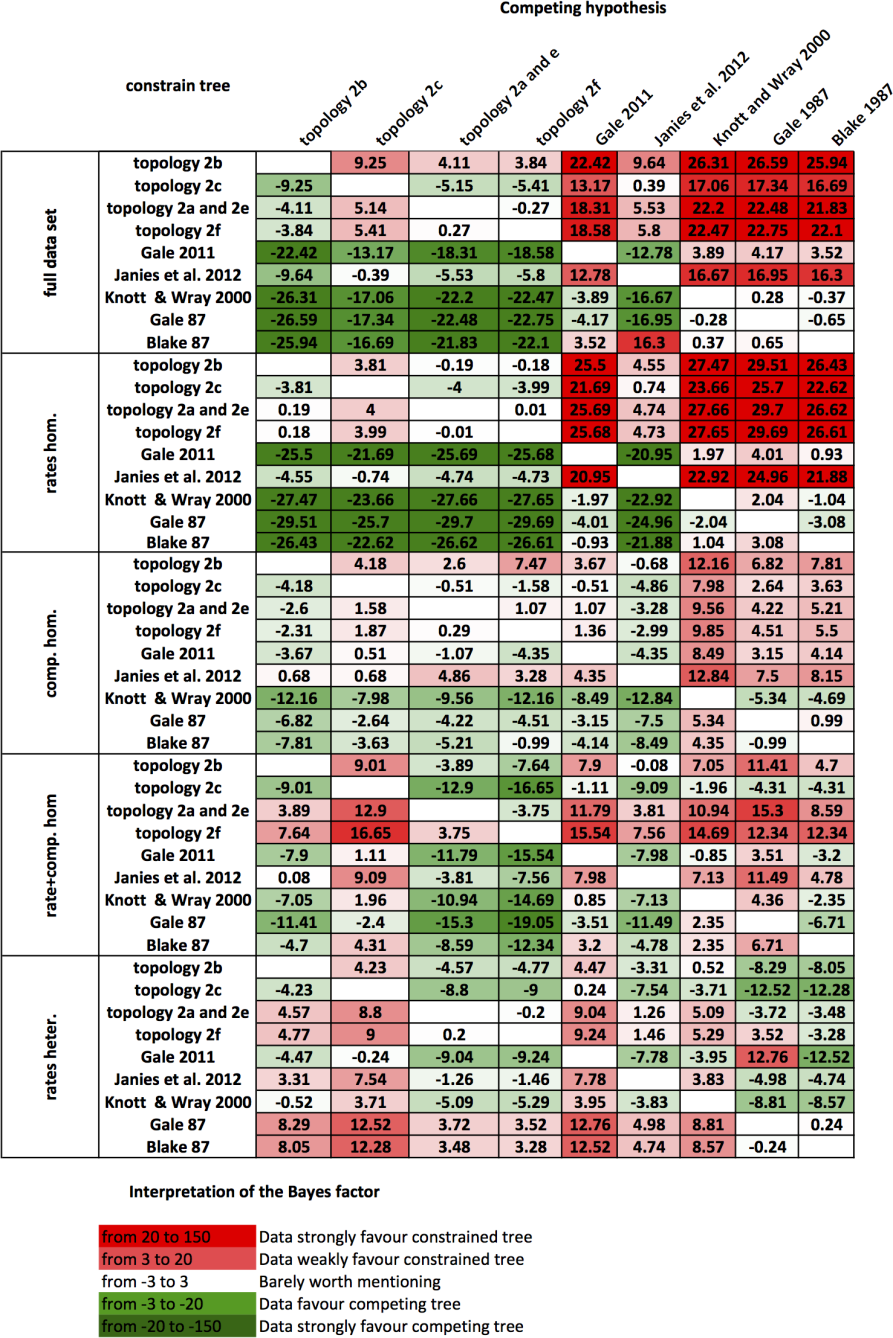
Bayes Factor support for the alterative topologies in the different partitions. Supports are coded according to [45]. Rates hom. = homogeneous rates partition; comp. hom. = compositionally homogeneous partition; rates+comp. hom. = homogeneous rates and composition partition; rates heter. = heterogeneous rates partition. In this table the red color implies that competing tree is rejected and the darker the red the stronger the evidence for rejection.

We also calculated the support in our seven data sets for the nine alternative phylogenetic hypotheses (Figs 1 and 2). The results (shown as a heat map in Fig 3) indicate that some of the previous published hypotheses i.e. Blake (1987), Gale (1987) Knott and Wray (2000) and Gale (2011) are not supported in any of the more reliable partitions (BF running from -21.7 in the rate homogenous data set to -19.05 in the composition+rate homogeneous data set). Significantly, the topologies of Gale (1987) and Blake (1987) increase their support in the less reliable rate heterogeneous partition and can thus be firmly rejected. Interestingly, the composition+rates homogenous data set is the only one consistently better supported compared to all other topologies, with BF values all positive and running from barely in favour (BF = 3.75) to values above 10.

## Discussion

### Phylogenetic signal and data partition reliability

The phylogenetic relationships of asteroids, especially the identity of the earliest branching clade, is a question that should be solvable using molecular data, but previous attempts have come to different solutions and no clear consensus has emerged. The main reason of this lack of consensus appears to be the small amount of phylogenetic signal that can be easily swamped by a combination of systematic and stochastic error.

The comparison of phylogenies obtained using differently fitting substitution models and different partitions has previously been used to explore the influence of non-phylogenetic signal on phylogenetic analyses [27, 29, 41–43]. The logic behind this approach is that unreliable partitions and poorly fitting models of evolution have a much greater chance of supporting artifactual topologies than reliable partitions and better fitting models. This is clearly demonstrated in our data by the fact that only the more reliable partitions find the expected relationships amongst the other echinoderm classes (Table 1). Following this logic, the increase of support we observe in the unreliable rate heterogeneous partition for the topologies of Blake 1987, Gale 1987, Knott and Wray 2000 and Gale 2011 shows that the strongest support for these comes from non-phylogenetic signal. By the same reasoning, all our results from the more reliable partitions analysed using the best fitting model, indicates that there is a strong phylogenetic signal supporting the Velatida as sister group to other asteroids, in keeping with Janies’ [8] findings. Only the most unreliable rate heterogeneous partition fails to support this topology.

A good example of how the ratio between noise and good phylogenetic signal is an issue that needs careful consideration comes when considering the relationships of Spinulosida to Forcipulata. The tree obtained from the full dataset, applying the GTR-Γ model, pairs Spinulosida and Forcipulata as a monophyletic group (Fig 2B). If the monophyly of this clade is genuine and not a phylogenetic artefact, we should see support for this clade increase in partitions with decreased compositional heterogeneity. Yet trees obtained under the best fitting model (CAT-GTR-Γ), non-homogeneous model (CATbp) and from compositional homogeneous data sets all fail to support the pairing of Spinulosida plus Forcipulata, indicating that the monophyly of this clade is most likely the result of non-phylogenetic signal.

**Fig 4:**
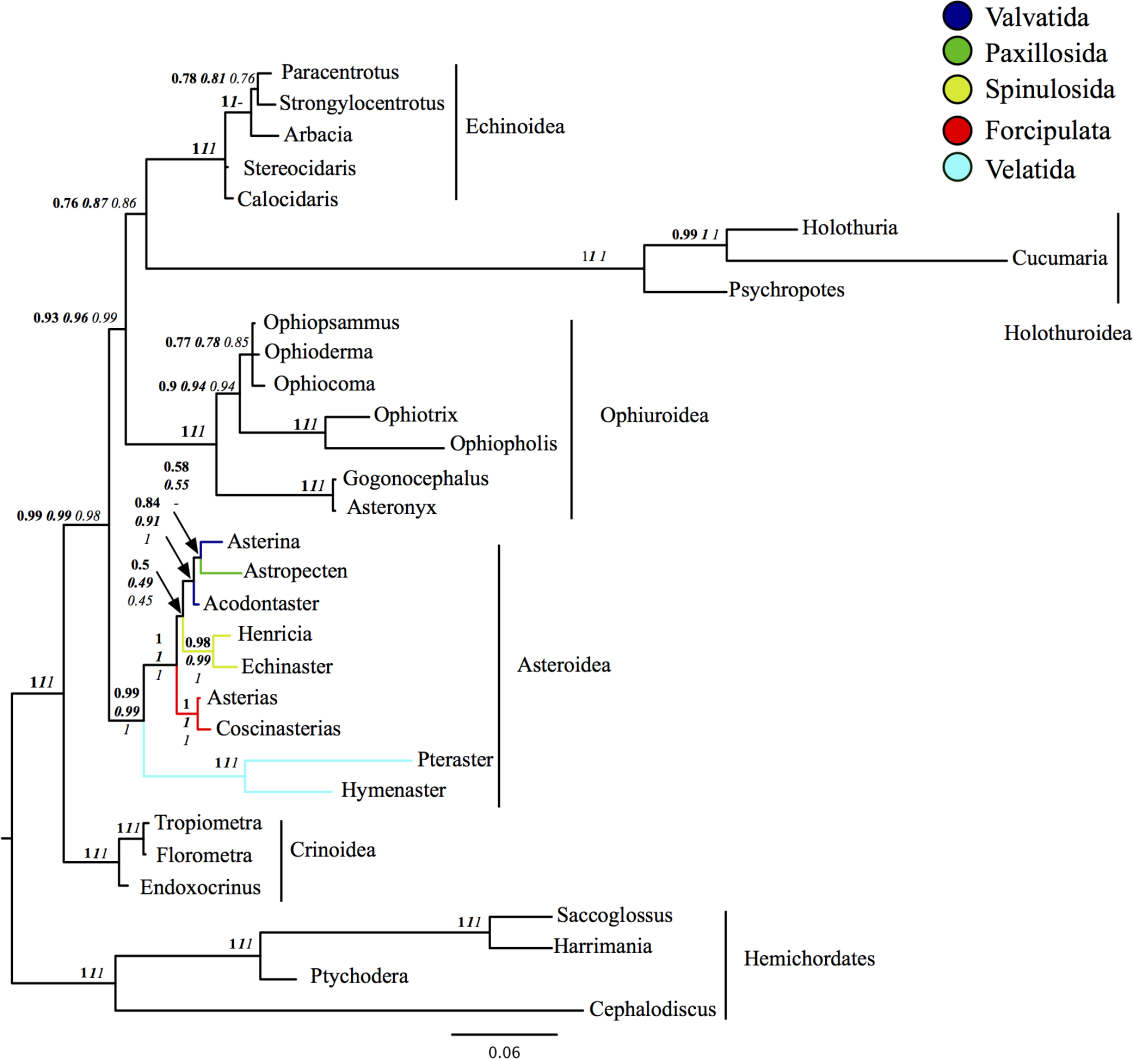
Resultant tree from analysis of the homogenous rates and compositional data set under CAT-GTR-Γ. Support at the nodes indicates posterior probability under CAT-GTR- Γ (bold), posterior probability GTR- Γ (italic bold) and maximum likelihood abayes bootstrap under GTR- Γ (italic).

The most reliable data sets are likely to be those minimizing rate and compositional heterogeneity and only these recover the well-established relationships in other echinoderm classes (Tables 1 and Table C in S1 File). The composition+rates homogeneous partition (Fig 2F and Fig 4) best meets all three criteria. Furthermore, our BF calculations suggest that overall the topology obtained from this data set is the best supported of all the topologies found (Fig 3). Based on these observations the tree in Figs 2F and 4 is taken as our best-supported topology.

While the reconstruction of secure and robust trees remains a major challenge for phylogeneticists where data are far from ideal, a more considered approach to rate and compositional heterogeneity in gene sequence data combined with the testing of competing hypotheses within a Bayesian framework, provide the best way forward, as demonstrated here.

### Morphological implications

**Fig 5:**
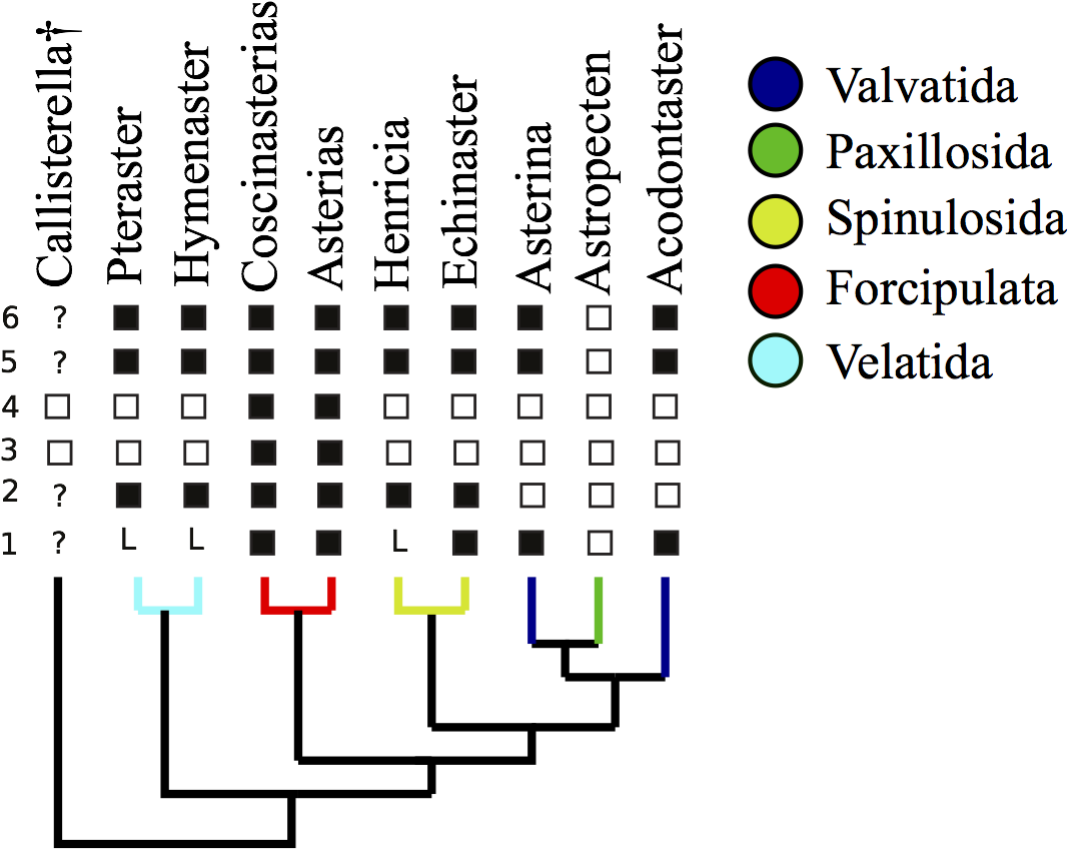
Key morphological characters relevant to asteroid rooting mapped onto our best-supported molecular phylogenetic tree. 1, planktotrophic larval development; ☐ to bipinnaria stage; ■to brachiolaria stage; L—lecithotrophic (no planktotrophic larval stage). 2, suckered tube feet; ☐ absent; ■present. 3, pedicellariae; ☐ simple valves; ■ complex, crossed pedicellarae with basal element. 4, oral frame; ☐ ambulacral; ■ adambulacral. 5, eversible stomach; ☐ absent; ■ present. 6, anus; ☐ absent; ■ present.

Our analysis of molecular data allows us to confidently reject topologies that place either Paxillosida or Forcipulata as sister group to other asteroids, as favoured by morphological studies [2–4, 12]. This implies a radical rethink of the body plan evolution of asteroids. Traditionally the Paxillosida had been seen as the most primitive of extant starfish because their larvae do not develop to the brachiolaria stage, they lack suckered tube-feet, they have a simple digestive system often lacking an anus and with a stomach that cannot be everted, and they have a relatively simple organization of their ambulacral-adambulacral skeleton [2–3]. Blake [4–5, 12] however, has argued that the paxillosids are highly specialised starfishes that have secondarily lost a number of characters, and our analysis supports the latter view (Fig 5). The presence of brachiolarian larvae (larvae with anterior arms and a sucker for attachment) in velatids, valvatids and forcipulatids must be seen as a shared plesiomorphy. As the brachiolarian larva is preceded developmentally by a bipinnaria larval stage [14, 44], and a bipinnaria stage is found in paxillosids, our tree implies that paxillosids must have truncated their development.

Suckered tube feet are fully developed only in Spinulosida and Forcipulata so their absence in Velatida, Paxillosida and many Valvatida is a shared plesiomorphy. Pedicellariae are present in some paxillosids, valvatids and forcipulatids but are often wanting; they are absent in spinulosids and velatids. Forcipulata possess complex, derived pedicellariae that are pedunculate [3] which contrasts with the more simple pedicellariae in paxillosids and valvatids. The fact that simple pedicellariae also occur in stem group asteroids [3] indicates that velatids have secondarily lost pedicellariae.

Gale [3] lists a number of features of the ambulacral-adambulacral skeleton in paxillosids that he considers primitive. Blake [4] also noted that paxillosids and many velatids had a primitive pattern in which adambulacrals are strongly overlapping with large muscles linking successive ambulacrals and with ambulacrals with long heads. However, in Gale’s [3] cladogram there is only a single characters relating to this complex that places paxillosids as primitive and that is the symmetry of the processes on ambulacral ossicles for adambulacral attachment. This is best explained as a retained plesiomorphy that other asteroids have lost through evolution. Finally, the oral frame of paxillosids and velatids has a similar organization, both having prominent narrow elongate oral plates, which must be plesiomorphic for crown group asteroids.

Sadly, from a morphologist’s viewpoint, optimizing the characters in Gale [3] onto our best-supported molecular tree identifies no reliable morphological traits that can be considered as unambiguously supporting a Valvatida-Paxillosida-Forcipulata-Spinulosida clade. Of the 15 characters separating the two clades all are either homoplasious or autapomorphies of Pterasteridae. For the present our best evidence for higher asteroid relationships must come from molecular data.

## Supporting Information

S1 Dataset (PHY)

S1 Fig CAT-GTR+ Γ tree of the full data set. (TIFF)

S2 Fig GTR+ Γ tree of the full data set. (TIFF)

S3 Fig CAT-BP+ Γ tree of the full dataset. (TIFF)

S4 Fig GTR+ Γ tree of the homogenous rates data set. (TIFF)

S5 Fig GTR+ Γ tree of the heterogeneous rates data set. (TIFF)

S6 Fig GTR+ Γ tree of the homogenous compositional data set. (TIFF)

S7 Fig GTR+ Γ tree of the homogenous rate+compositional data set. (TIFF)

S8 Fig Maximum likelihood GTR+ Γ tree of the full data set. (TIFF)

S9 Fig Maximum likelihood GTR+ Γ tree of the homogenous rates data set. (TIFF)

S10 Fig Maximum likelihood GTR+ Γ tree of the heterogeneous rates data set. (TIFF)

S11 Fig Maximum likelihood GTR+ Γ tree of homogenous compositional data set. (TIFF)

S12 Fig Maximum likelihood GTR+ Γ tree homogenous rate+compositional data set. (TIFF)

S1 File Supporting tables. Table A, List of taxa and gene sequences used in this study. Consensus genus sequences were constructed from these. Table B, Result of the 12-fold Bayesian cross-validation. Positive scores indicate that the model compared is better than the reference model (CATGTR). Table C, Statistical comparison of the compositional heterogeneity in the various partitions. Z-scores quantify the amount of compositional heterogeneity (the greater the z-score the higher the compositional heterogeneity), p-values indicate the statistical significance of test with * indicating significant values. Compositional heterogeneity clearly is not an issue in the compositional+rates homogenous data set based on these tests. Table D, Results of the posterior predictive estimation of the homoplasy in the nuclear and nuclear+mitochondrial genes data sets. Although the p-values are significant for both data sets, the observed homoplasy is higher in the nuclear+mitochondrial data set.
(DOCX)
