# Supplementary figures and images for: Phylogenetic Signal Dissection Identifies the Root of Starfishes
Source: PLoS One. 2015 May 8;10(5):e0123331. doi: 10.1371/journal.pone.0123331 (PMC4425436; doi:10.1371/journal.pone.0123331)

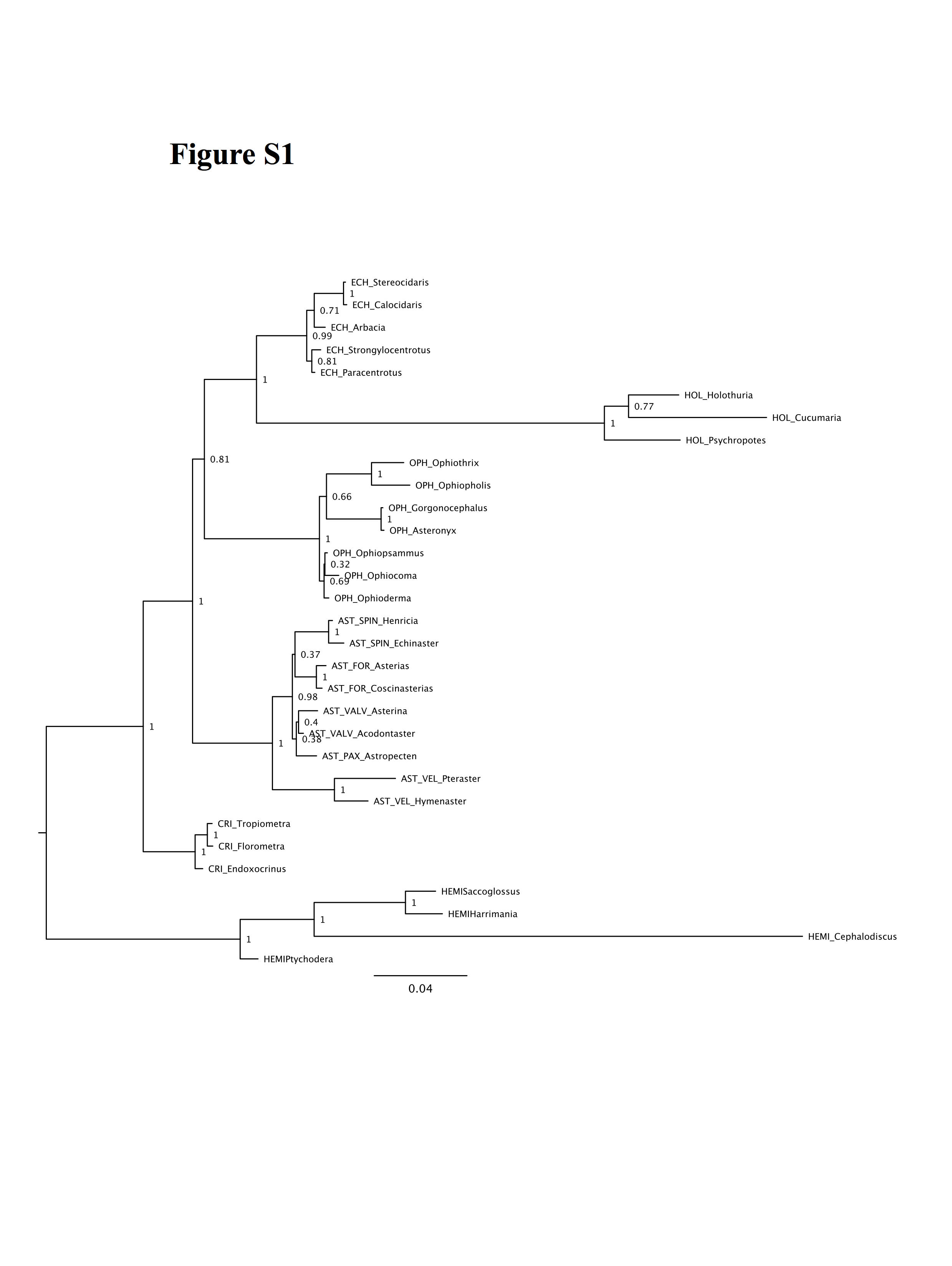

Supplement: S1 Fig — (TIFF) [file pone.0123331.s002.tiff]

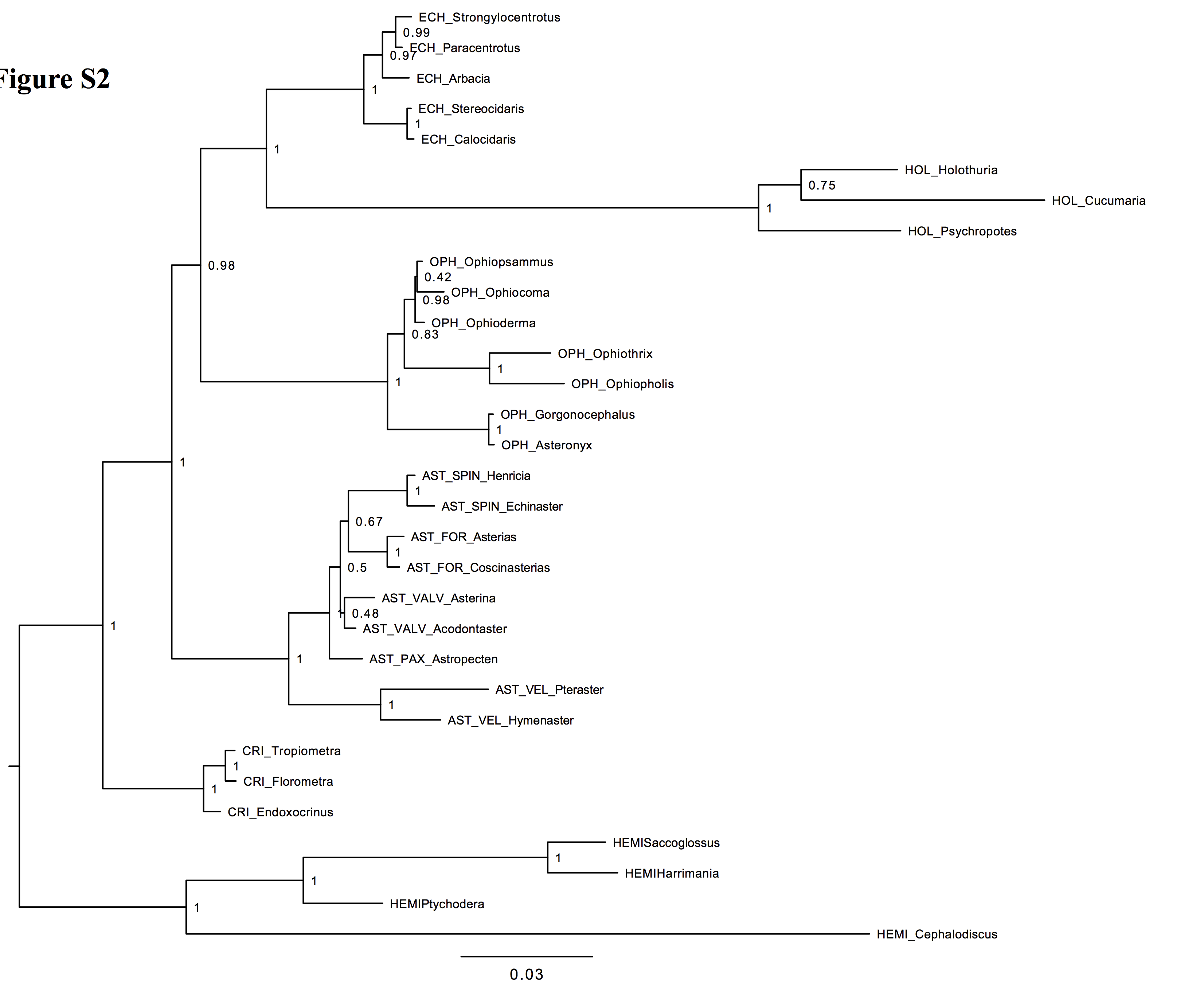

Supplement: S2 Fig — (TIFF) [file pone.0123331.s003.tiff]

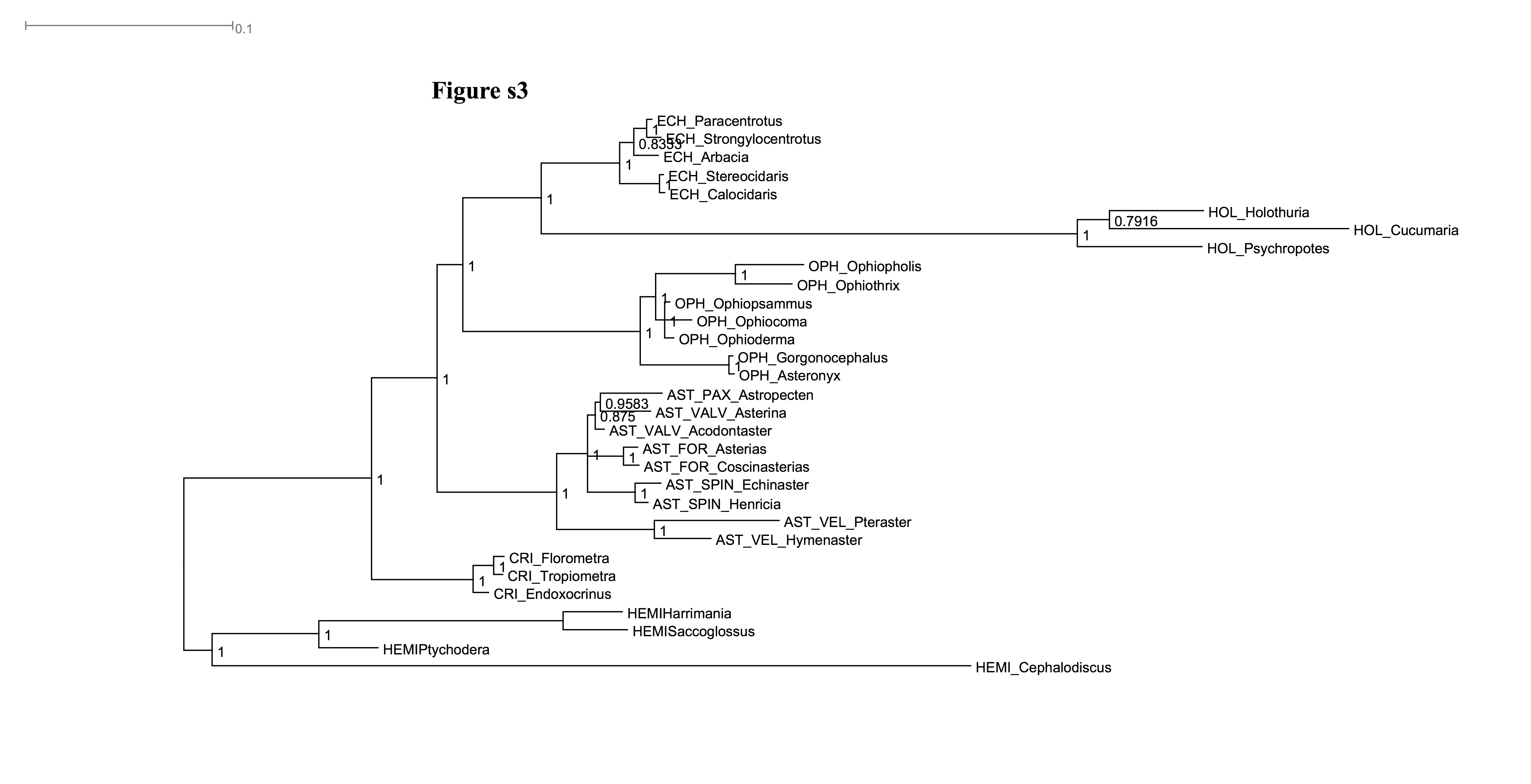

Supplement: S3 Fig — (TIFF) [file pone.0123331.s004.tiff]

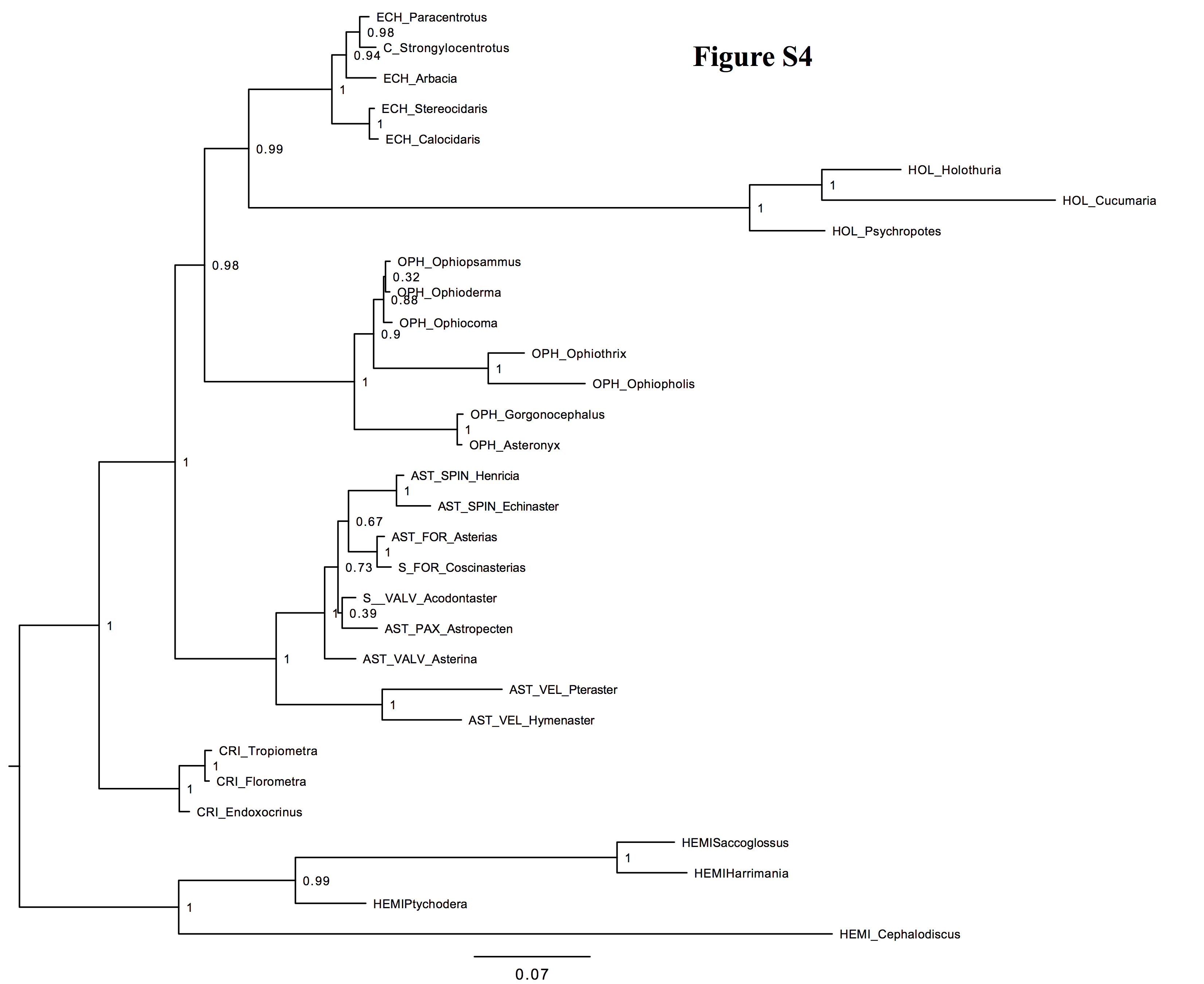

Supplement: S4 Fig — (TIFF) [file pone.0123331.s005.tiff]

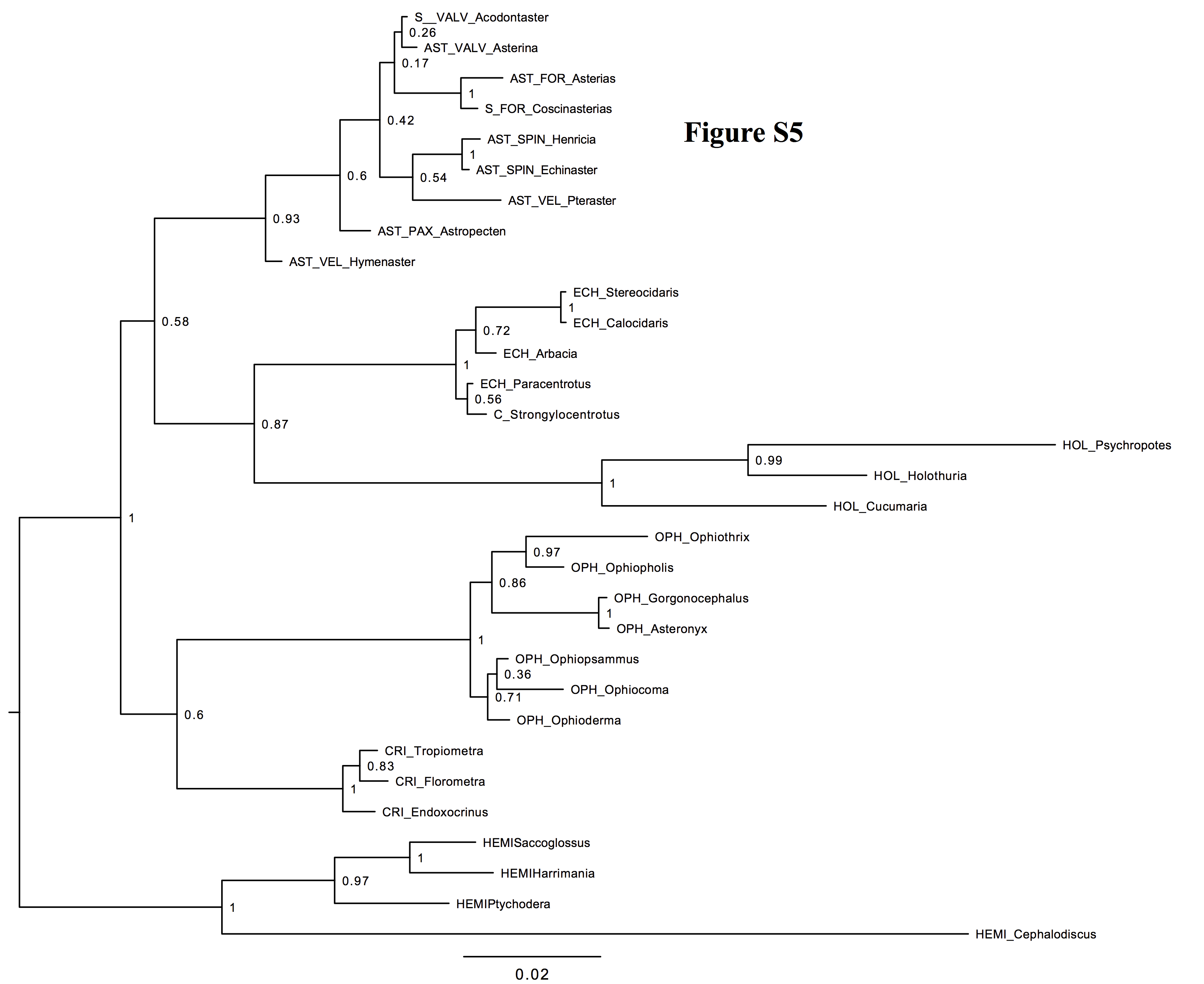

Supplement: S5 Fig — (TIFF) [file pone.0123331.s006.tiff]

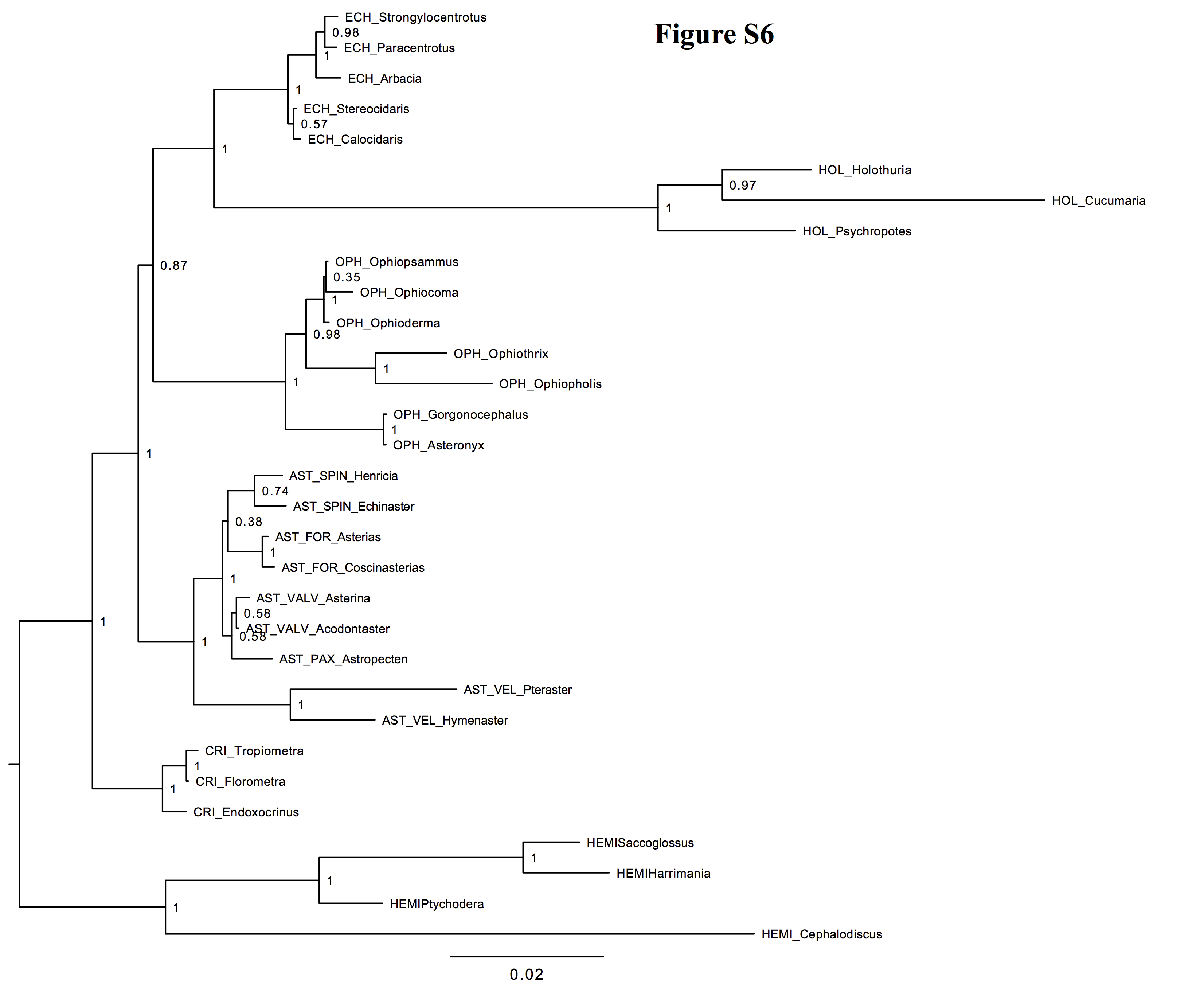

Supplement: S6 Fig — (TIFF) [file pone.0123331.s007.tiff]

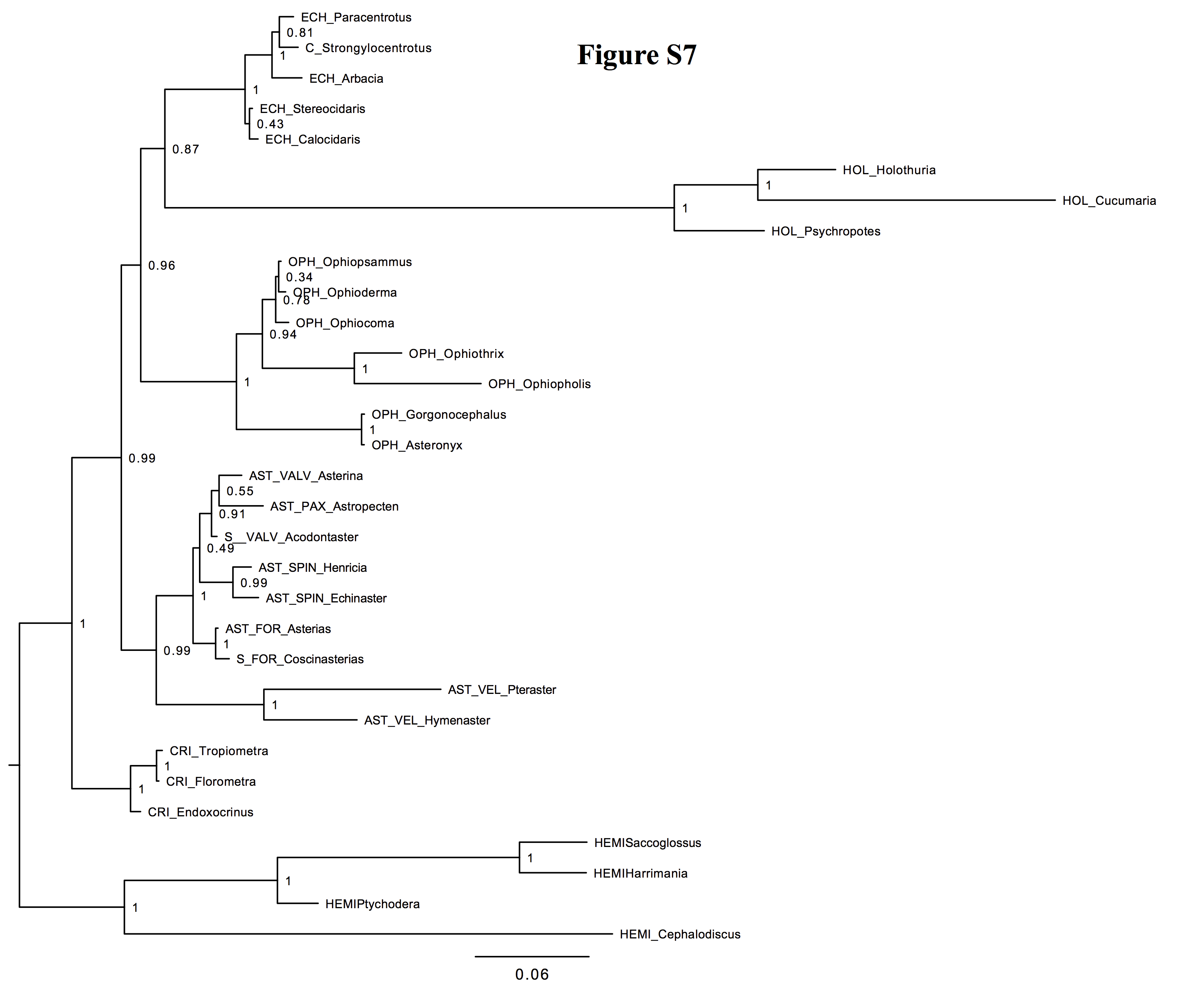

Supplement: S7 Fig — (TIFF) [file pone.0123331.s008.tiff]

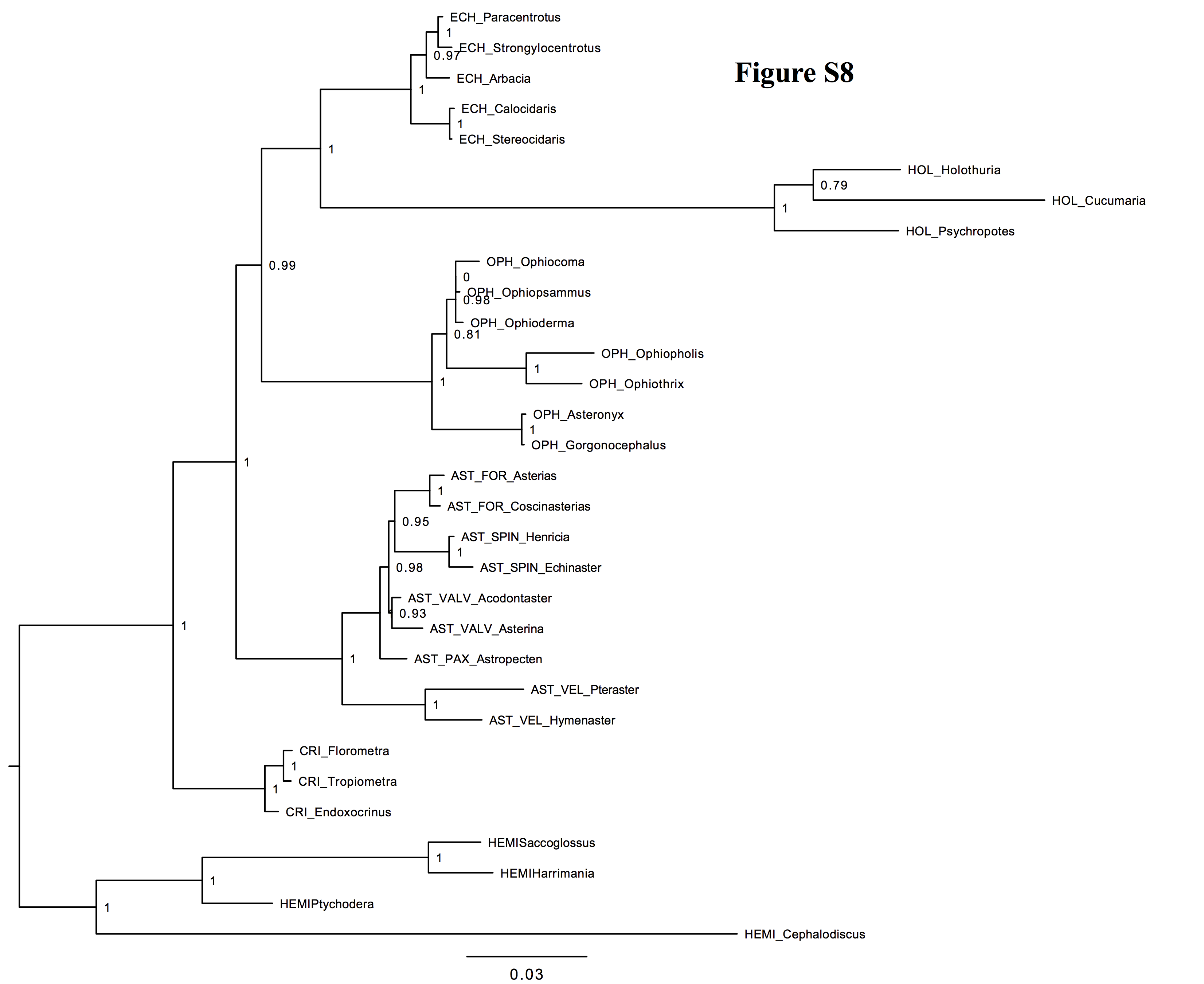

Supplement: S8 Fig — (TIFF) [file pone.0123331.s009.tiff]

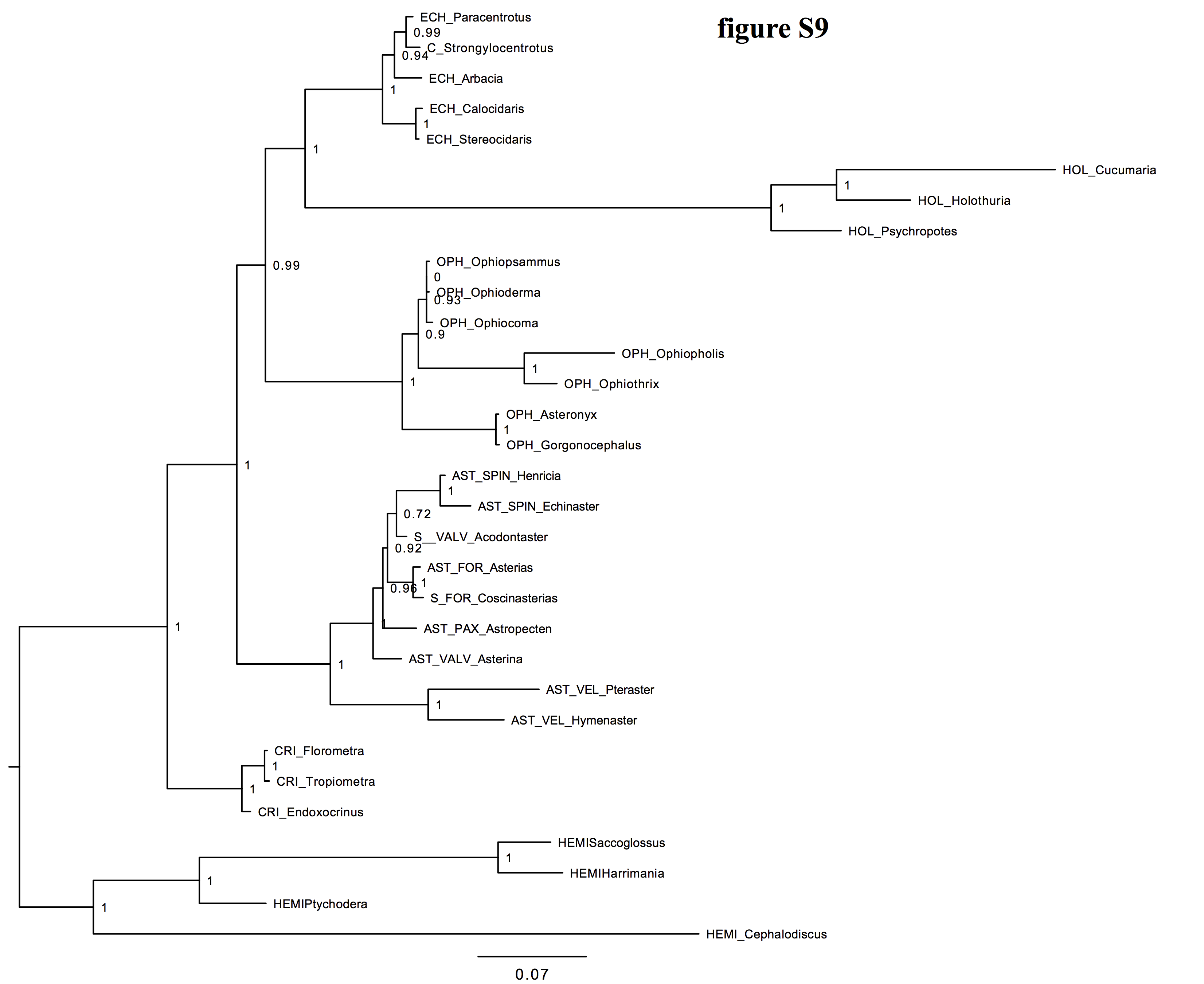

Supplement: S9 Fig — (TIFF) [file pone.0123331.s010.tiff]

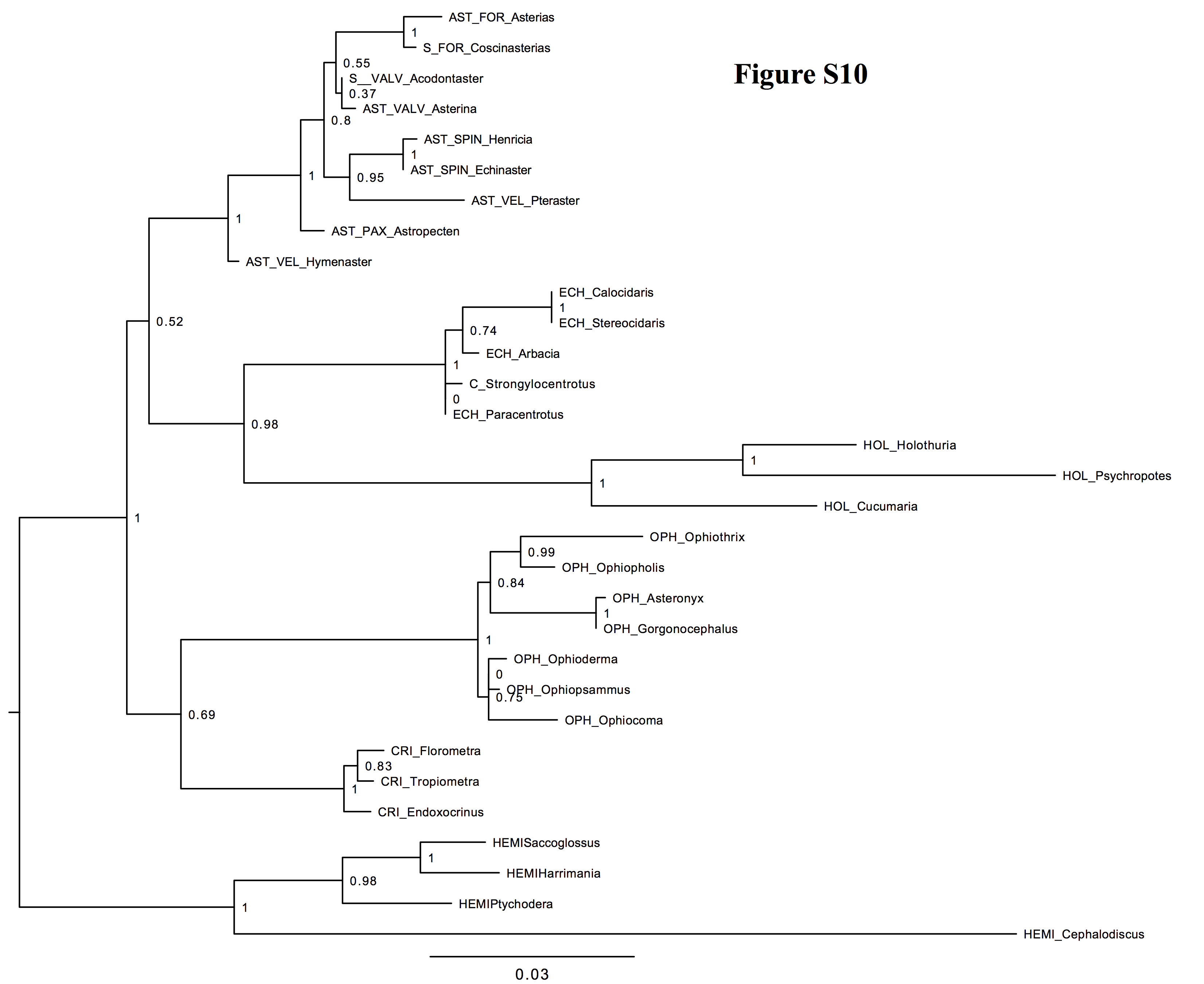

Supplement: S10 Fig — (TIFF) [file pone.0123331.s011.tiff]

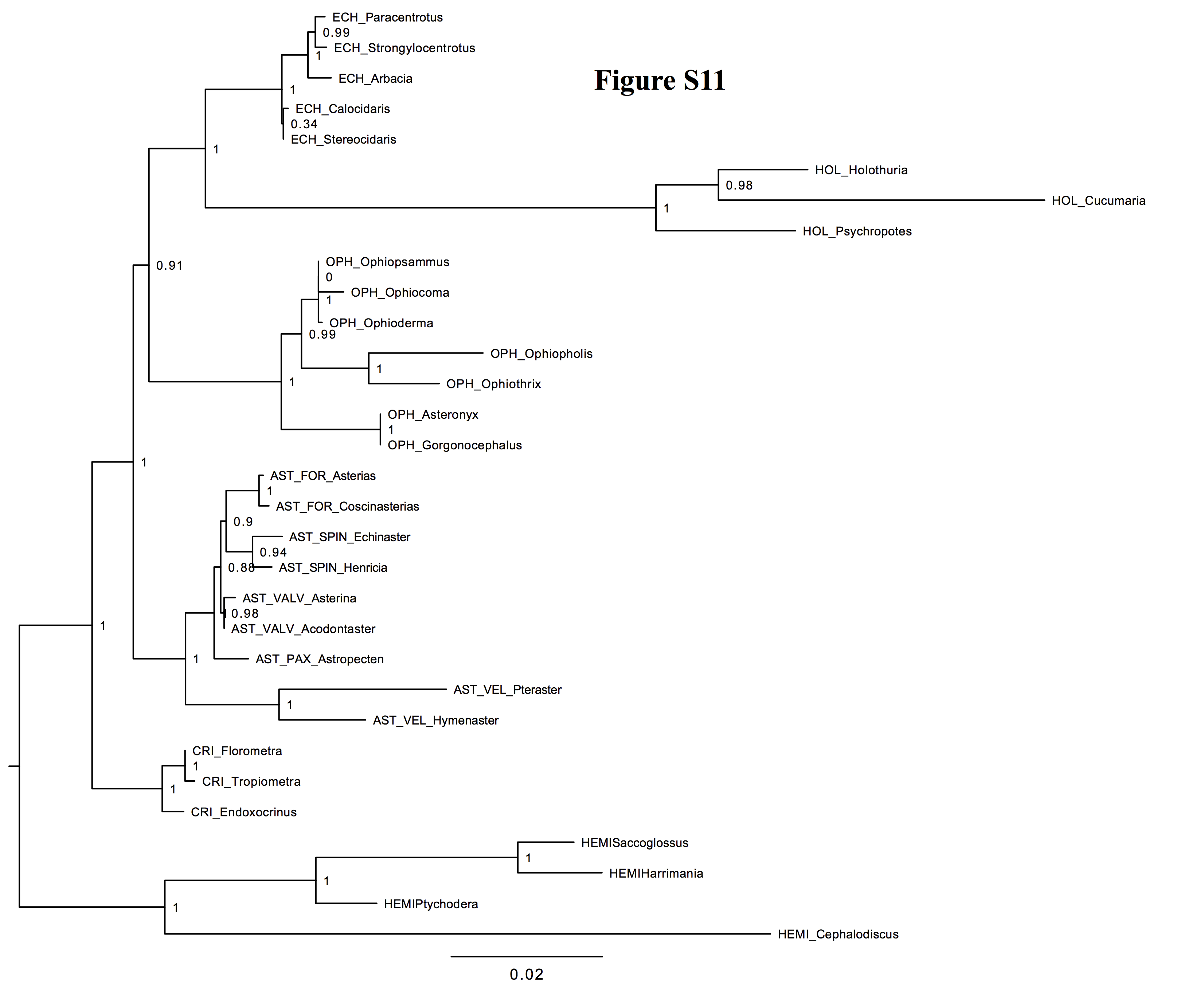

Supplement: S11 Fig — (TIFF) [file pone.0123331.s012.tiff]

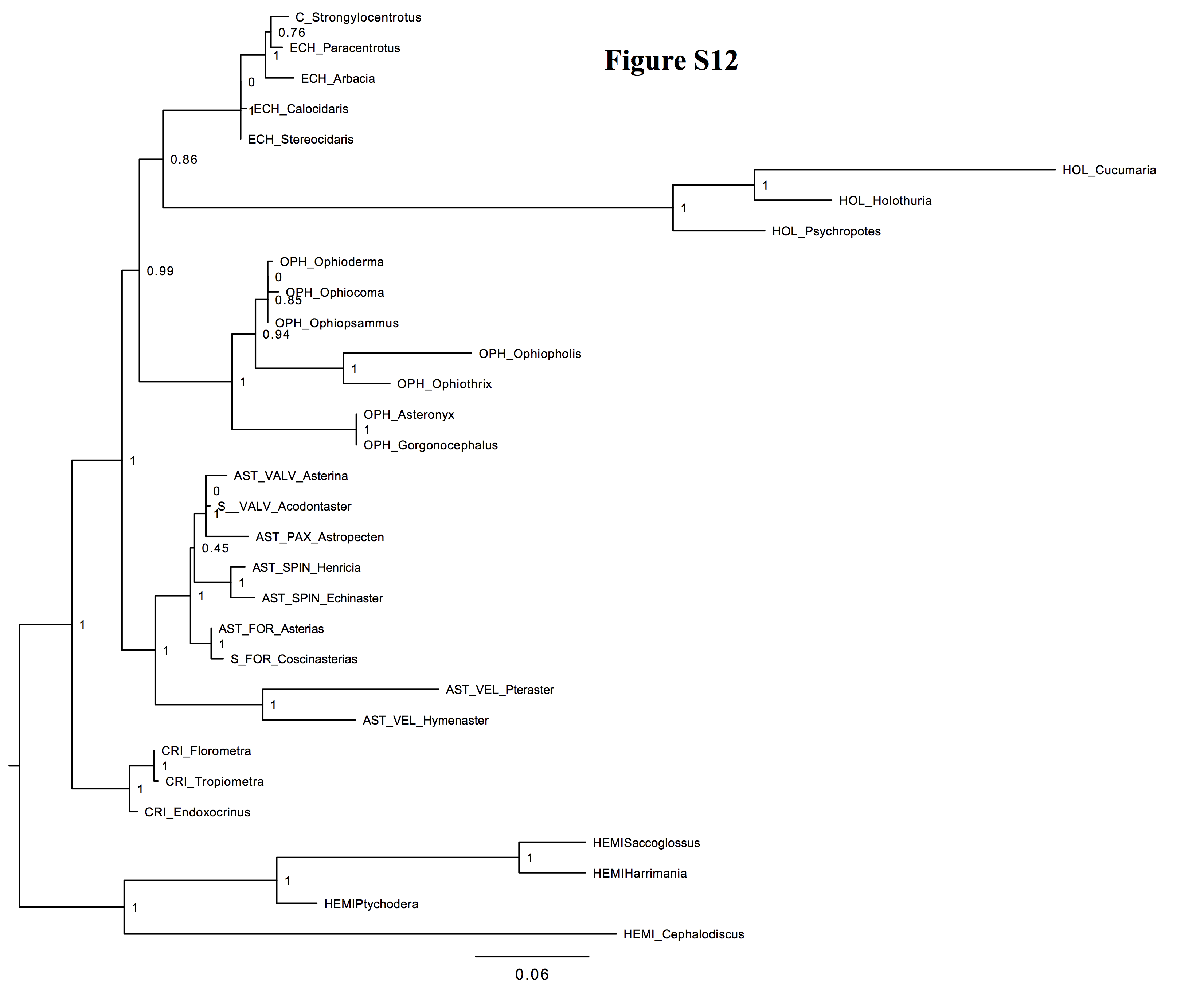

Supplement: S12 Fig — (TIFF) [file pone.0123331.s013.tiff]
