# Supplementary material for: Phylogenetic Signal Dissection Identifies the Root of Starfishes
Source: PLoS One. 2015 May 8;10(5):e0123331. doi: 10.1371/journal.pone.0123331 (PMC4425436; doi:10.1371/journal.pone.0123331)
Supplement: S1 File — Table A, List of taxa and gene sequences used in this study. Consensus genus sequences were constructed from these. Table B, Result of the 12-fold Bayesian cross-validation. Positive scores indicate that the model compared is better than the reference model (CATGTR). Table C, Statistical comparison of the compositional heterogeneity in the various partitions. Z-scores quantify the amount of compositional heterogeneity (the greater the z-score the higher the compositional heterogeneity), p-values indicate the statistical significance of test with * indicating significant values. Compositional heterogeneity clearly is not an issue in the compositional+rates homogenous data set based on these tests. Table D, Results of the posterior predictive estimation of the homoplasy in the nuclear and nuclear+mitochondrial genes data sets. Although the p-values are significant for both data sets, the observed homoplasy is higher in the nuclear+mitochondrial data set. (DOCX) [file pone.0123331.s014.docx]

**Phylogenetic signal dissection identifies the root of Starfishes**

Roberto Feuda and Andrew B. Smith

Table A

| **Phylum** | **Class** | **Genus** | **Species** | **18S rRNA** | **28S rRNA** |
| --- | --- | --- | --- | --- | --- |
| Hemichordata | Enteropneusta | Saccoglossus | kowalevskii | L28054 | AF212175 |
|  |  | Harrimania | planktophilus | AF236799 | AF212173 |
|  |  | Ptychodera | flava | AF278681 | AF212176 |
|  | Pterobranchia | Cephalodiscus | gracilis | AF236798 | AF212172 |
| Echinodermata | Crinoidea | Florometra | seratissima | DQ060789 | AF22168 |
|  |  | Tropiometra | carinata | GU327941 |  |
|  |  |  | afra |  | GU327978 |
|  | Asteroidea | Asterias | forbesi | DQ060776 | AF212169 |
|  |  | Coscinasterias | acutispina | AB125601 | AJ225847 |
|  |  |  | muricata |  | DQ060008, DQ273704, DQ297117 |
|  |  | Pteraster | militaris | DQ060814 | DQ060030, DQ273719 |
|  |  | Hymenaster | pellucidus | DQ060792 | DQ029081, DQ060016, DQ273700 |
|  |  | Astropecten | irregularis | Z80949 | AJ225837 |
|  |  |  | polycanthus | DQ060782 | DQ060007 |
|  |  | Henricia | sanguinolenta | DQ077936 |  |
|  |  |  | sp. |  | AJ225845, DQ273707, DQ297118, DQ060015 |
|  |  | Echinaster | sentus | DQ060786 | DQ060012 |
|  |  |  | sepositus |  | AJ225844, AF088831 |
|  |  | Asterina | gibbosa | AF088801 | AF088839, AJ225840 |
|  |  |  | miniata |  | DQ060004 |
|  |  | Acodontaster | conspicuus | DQ060774 | DQ060003, DQ273703 |
|  | Ophiuroidea | Asteronyx | sp. | DQ060809 | DQ029071, DQ060005, DQ273716 |
|  |  | Gorgonocephalus | eucnemis | DQ060790 | DQ029072, DQ060014, DQ297119 |
|  |  | Ophioderma | cenereum | AY859645 | AY859643 |
|  |  |  | brevispinum | DQ060803 | AY859643 |
|  |  |  | longicauda |  | AJ225819 |
|  |  | Ophiothrix | oerstedii | DQ060808 | DQ297128, DQ029079, DQ273715 |
|  |  |  | fragilis |  | AJ225820 |
|  |  | Ophiocoma | echinata | DQ060802 | DQ060020, DQ273709, DQ297122 |
|  |  | Ophiopsammus | maculata | DQ060807 | DQ029076, DQ060025, DQ273714, DQ297126 |
|  |  | Ophiopholis | aculeata | DQ060806 | AJ225836, DQ029078, DQ060024, DQ273713, DQ297127 |
|  | Echinoidea | Calocidaris | micans | DQ073782 | DQ073756 |
|  |  | Stereocidaris | excavatus | DQ073795 | DQ073772 |
|  |  | Arbacia | lixula | Z37514 | DQ073753 |
|  |  | Paracentrotus | lividus | AM981272 | AM981272 |
|  |  | Strongylocentrotus | purpuratus | L28055 | AF212171 |
|  | Holothuroidea | Psychropotes | longicauda | Z80956 | Z80946, DQ777094 |
|  |  | Cucumaria | sykion | Z80950 |  |
|  |  |  | salma |  | AF212170 |
|  |  | Holothuria | forskali | AY133470 | AJ225810 |
|  |  |  | leucospilota |  | DQ777093 |

Table B

| models compared | mean score +- stdev | #times model is best |
| --- | --- | --- |
|  |  |  |
| GTR versus CATGTR | -5.34083 +/- 3.54623 | 1 |
| CAT versus CATGTR | -1.47917 +/- 1.75753 | 2 |

Table C

|  | **full data set** | | **rates homogeneous** | | **rates heterogeneous** | | **comp. homogeneous** | | **comp+rates homogeneous** | |
| --- | --- | --- | --- | --- | --- | --- | --- | --- | --- | --- |
| **Taxa** | p-value | z-score | p-value | z-score | p-value | z-score | p-value | z-score | p-value | z-score |
| **HEMI_Saccoglossus** | 0* | 5.29 | 0.004* | 4.295 | 0.072 | 1.896 | 0.588 | -0.382 | 0.986 | -1.232 |
| **HEMI_Harrimania** | 0* | 6.877 | 0* | 5.992 | 0.218 | 0.944 | 0.411 | 0.099 | 0.853 | -0.889 |
| **HEMI_Ptychodera** | 0.802 | -0.828 | 0.874 | -0.89 | 0.654 | -0.468 | 0.595 | -0.46 | 0.6 | -0.515 |
| **HEMI_Cephalodiscus** | 0* | 15.894 | 0* | 15.688 | 0* | 5.952 | 0.028* | 2.329 | 0.213 | 0.661 |
| **CRI_Florometra** | 0* | 9.034 | 0.001* | 6.408 | 0.2 | 0.778 | 0.9 | -1.115 | 0.16 | 0.812 |
| **CRI_Tropiometra** | 0.079 | 1.64 | 0.006* | 4.639 | 0.781 | -0.802 | 0.822 | -0.94 | 0.913 | -0.923 |
| **CRI_Endoxocrinus** | 0.002* | 5.703 | 0.004* | 3.936 | 0.018* | 2.452 | 0.978 | -1.183 | 1 | -0.911 |
| **AST_FOR_Asterias** | 0.016* | 2.76 | 0.096 | 1.362 | 0.072 | 1.329 | 0.921 | -1.054 | 0.793 | -0.762 |
| **AST_FOR_Coscinasterias** | 0.03* | 2.315 | 0.074 | 1.458 | 0.254 | 0.425 | 0.794 | -0.775 | 0.746 | -0.768 |
| **AST_PAX_Astropecten** | 0.146 | 0.937 | 0.083 | 1.445 | 0.4 | 0.203 | 0.843 | -0.992 | 0.693 | -0.57 |
| **AST_VEL_Pteraster** | 0.093 | 1.303 | 0.491 | -0.326 | 0.236 | 0.674 | 0.737 | -0.627 | 0.813 | -0.874 |
| **AST_VEL_Hymenaster** | 0.525 | -0.24 | 0.246 | 0.446 | 0.854 | -0.941 | 0.276 | 0.601 | 0.44 | -0.104 |
| **AST_SPIN_Henricia** | 0.002* | 4.916 | 0.075 | 1.584 | 0* | 3.862 | 0.936 | -1.086 | 0.64 | -0.561 |
| **AST_SPIN_Echinaster** | 0.874 | -1.104 | 0.232 | 0.548 | 1 | -1.904 | 0.262 | 0.474 | 0.353 | 0.255 |
| **AST_VALV_Asterina** | 0.055 | 1.934 | 0.009* | 3.917 | 0.818 | -0.822 | 0.695 | -0.657 | 0.9 | -1.009 |
| **AST_VALV_Acodontaster** | 0.044* | 2.063 | 0.131 | 1.029 | 0.272 | 0.616 | 0.567 | -0.323 | 0.82 | -0.866 |
| **OPH_Asteronyx** | 0.039* | 2.557 | 0.001* | 4.834 | 0.836 | -0.926 | 0.723 | -0.655 | 0.78 | -0.841 |
| **OPH_Ophiopsammus** | 0.425 | 0.021 | 0.01* | 3.4 | 0.89 | -0.988 | 0.517 | -0.148 | 0.573 | -0.379 |
| **OPH_Ophioderma** | 0* | 6.364 | 0.001* | 5.177 | 0.636 | -0.6 | 0.73 | -0.7 | 0.306 | 0.318 |
| **OPH_Ophiopholis** | 0.02* | 3.163 | 0.009* | 4.056 | 0.763 | -0.747 | 0.865 | -1.1 | 0.966 | -1.164 |
| **OPH_Ophiocoma** | 0* | 5.601 | 0.018* | 3.013 | 0.018* | 2.09 | 0.964 | -1.068 | 0.466 | -0.094 |
| **OPH_Ophiothrix** | 0* | 5.657 | 0.051 | 1.972 | 0.054 | 2.225 | 0.957 | -1.15 | 0.646 | -0.594 |
| **OPH_Gorgonocephalus** | 0.002* | 6.439 | 0* | 5.735 | 0.109 | 1.25 | 0.836 | -0.824 | 0.866 | -0.899 |
| **ECH_Calocidaris** | 0.895 | -0.933 | 0.724 | -0.656 | 0.963 | -1.219 | 0.822 | -0.871 | 0.58 | -0.443 |
| **ECH_Arbacia** | 0.202 | 0.621 | 0.482 | -0.184 | 0.145 | 0.936 | 0.744 | -0.754 | 0.6 | -0.447 |
| **ECH_Strongylocentrotus** | 0.062 | 1.571 | 0.649 | -0.579 | 0.036* | 2.603 | 0.432 | -0.158 | 0.826 | -0.823 |
| **ECH_Stereocidaris** | 0.974 | -1.18 | 0.784 | -0.797 | 0.909 | -1.255 | 0.829 | -0.928 | 0.606 | -0.437 |
| **ECH_Paracentrotus** | 0.69 | -0.577 | 0.795 | -0.802 | 0.927 | -1.061 | 0.617 | -0.48 | 0.42 | -0.062 |
| **HOL_Cucumaria** | 0.723 | -0.709 | 0.877 | -0.951 | 0.8 | -0.874 | 0.9 | -0.85 | 0.82 | -0.844 |
| **HOL_Holothuria** | 0.72 | -0.65 | 0.272 | 0.281 | 0.454 | -0.067 | 0.794 | -0.801 | 0.96 | -1.126 |
| **HOL_Psychropotes** | 0.058 | 1.932 | 0.068 | 1.791 | 0* | 5.085 | 0.624 | -0.475 | 0.72 | -0.686 |

**Table D**

| PPA for saturation | | |
| --- | --- | --- |
|  | observed homoplasy | p-values |
| nuclear+mitochondrial genes | 0.220078 +/- 0.00674892 | 0.0114943 |
| nuclear genes | 0.180744 +/- 0.0071688 | 0.0208877 |
